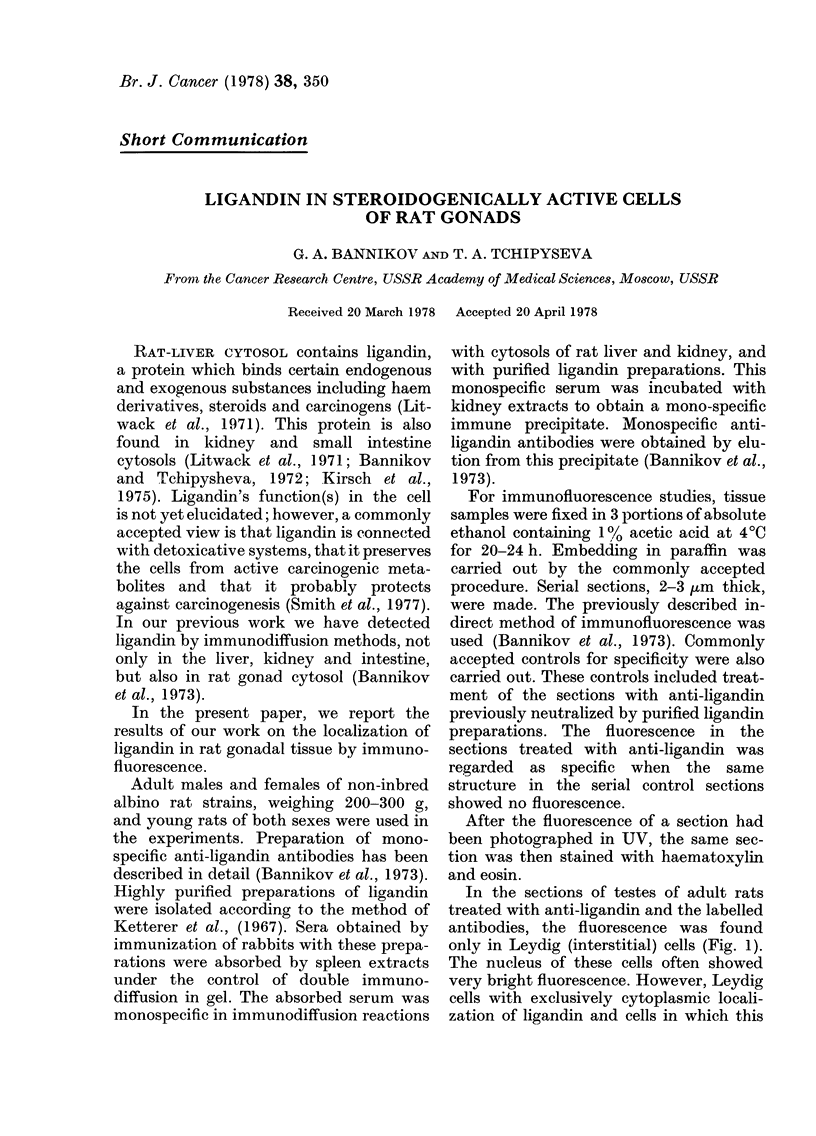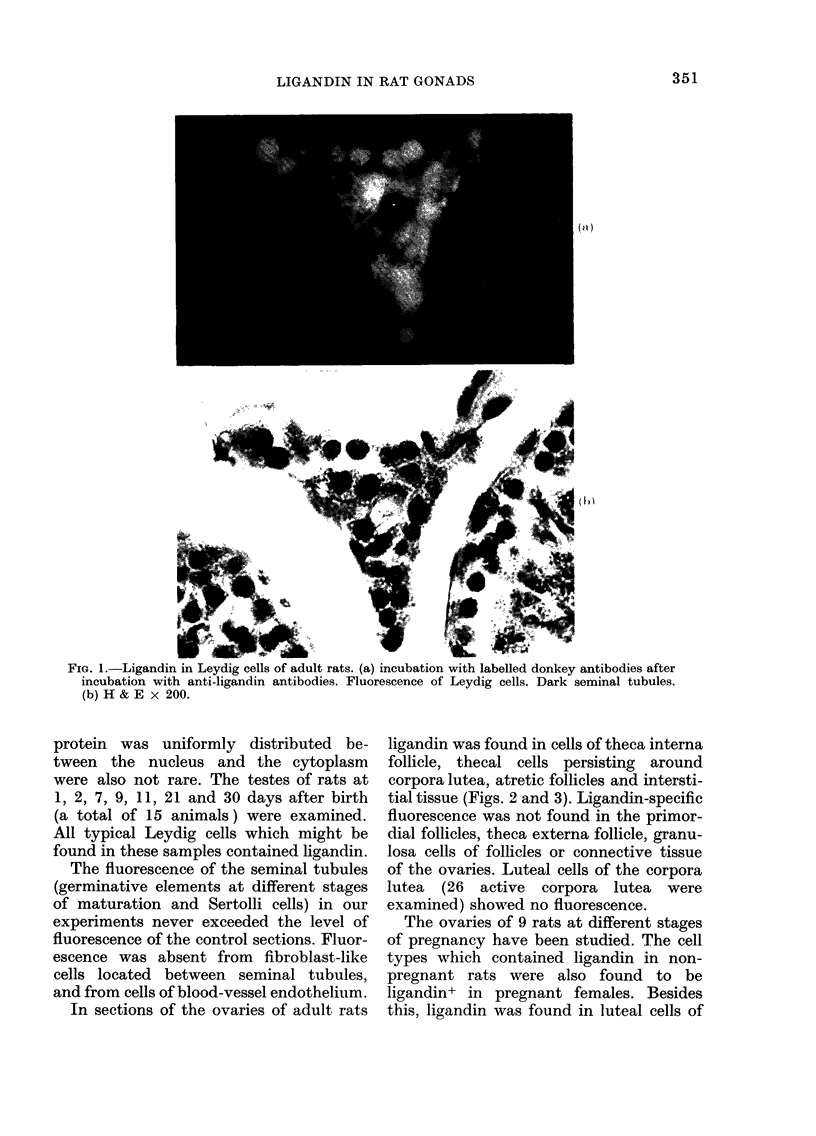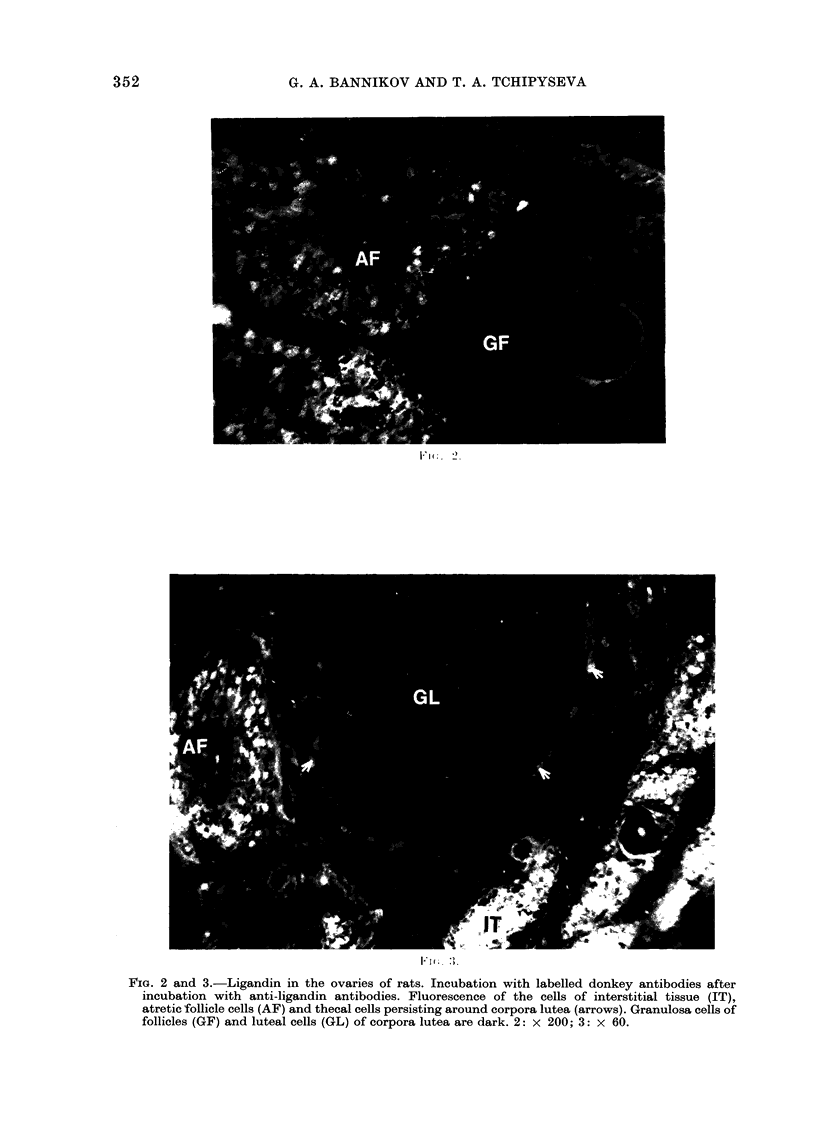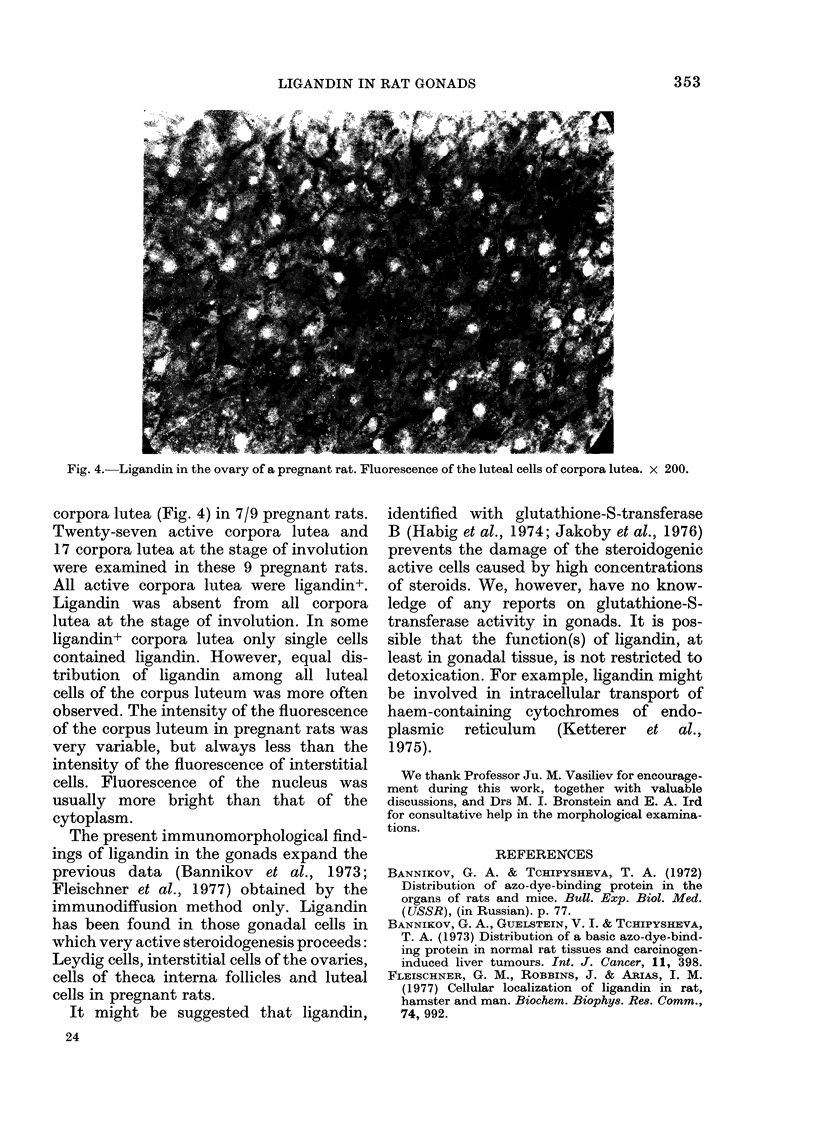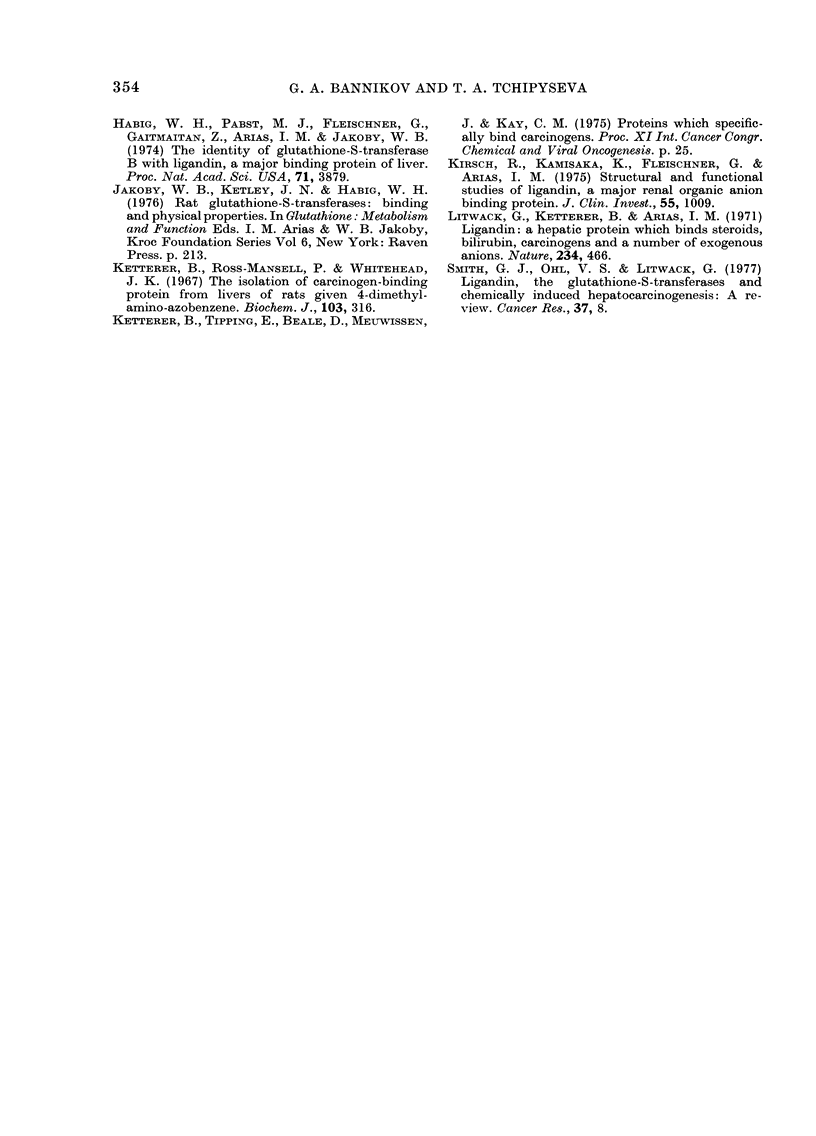# Ligandin in steroidogenically active cells of rat gonads.

**DOI:** 10.1038/bjc.1978.210

**Published:** 1978-08

**Authors:** G. A. Bannikov, T. A. Tchipyseva

## Abstract

**Images:**


Br. J. Cancer (1978) 38, 350

### Short Communication

### LIGANDIN IN STEROIDOGENICALLY ACTIVE CELLS OF RAT GONADS

G. A. BANNIKOV AND T. A. TCHIPYSEVA

From the Cancer Research Centre, USSR Academy of Medical Sciences, Moscow, USSR

Received 20 March 1978 Accepted 20 April 1978

RAT-LIVER CYTOSOL contains ligandin, a protein which binds certain endogenous and exogenous substances including haem derivatives, steroids and carcinogens (Litwack *et al.*, 1971). This protein is also found in kidney and small intestine cytosols (Litwack *et al.*, 1971; Bannikov and Tchipysheva, 1972; Kirsch *et al.*, 1975). Ligandin's function(s) in the cell is not yet elucidated; however, a commonly accepted view is that ligandin is connected with detoxicative systems, that it preserves the cells from active carcinogenic metabolites and that it probably protects against carcinogenesis (Smith *et al.*, 1977). In our previous work we have detected ligandin by immunodiffusion methods, not only in the liver, kidney and intestine, but also in rat gonad cytosol (Bannikov *et al.*, 1973).

In the present paper, we report the results of our work on the localization of ligandin in rat gonadal tissue by immunofluorescence.

Adult males and females of non-inbred albino rat strains, weighing 200–300 g, and young rats of both sexes were used in the experiments. Preparation of monospecific anti-ligandin antibodies has been described in detail (Bannikov *et al.*, 1973). Highly purified preparations of ligandin were isolated according to the method of Ketterer *et al.*, (1967). Sera obtained by immunization of rabbits with these preparations were absorbed by spleen extracts under the control of double immunodiffusion in gel. The absorbed serum was monospecific in immunodiffusion reactions with cytosols of rat liver and kidney, and with purified ligandin preparations. This monospecific serum was incubated with kidney extracts to obtain a mono-specific immune precipitate. Monospecific antiligandin antibodies were obtained by elution from this precipitate (Bannikov *et al.*, 1973).

For immunofluorescence studies, tissue samples were fixed in 3 portions of absolute ethanol containing 1% acetic acid at 4°C for 20–24 h. Embedding in paraffin was carried out by the commonly accepted procedure. Serial sections, 2–3 $\mu$m thick, were made. The previously described indirect method of immunofluorescence was used (Bannikov *et al.*, 1973). Commonly accepted controls for specificity were also carried out. These controls included treatment of the sections with anti-ligandin previously neutralized by purified ligandin preparations. The fluorescence in the sections treated with anti-ligandin was regarded as specific when the same structure in the serial control sections showed no fluorescence.

After the fluorescence of a section had been photographed in UV, the same section was then stained with haematoxylin and eosin.

In the sections of testes of adult rats treated with anti-ligandin and the labelled antibodies, the fluorescence was found only in Leydig (interstitial) cells (Fig. 1). The nucleus of these cells often showed very bright fluorescence. However, Leydig cells with exclusively cytoplasmic localization of ligandin and cells in which this

FIG. 1.—Ligandin in Leydig cells of adult rats. (a) incubation with labelled donkey antibodies after incubation with anti-ligandin antibodies. Fluorescence of Leydig cells. Dark seminal tubules. (b) H & E × 200.

protein was uniformly distributed between the nucleus and the cytoplasm were also not rare. The testes of rats at 1, 2, 7, 9, 11, 21 and 30 days after birth (a total of 15 animals) were examined. All typical Leydig cells which might be found in these samples contained ligandin.

The fluorescence of the seminal tubules (germinative elements at different stages of maturation and Sertolli cells) in our experiments never exceeded the level of fluorescence of the control sections. Fluorescence was absent from fibroblast-like cells located between seminal tubules, and from cells of blood-vessel endothelium.

In sections of the ovaries of adult rats ligandin was found in cells of theca interna follicle, thecal cells persisting around corpora lutea, atretic follicles and interstitial tissue (Figs. 2 and 3). Ligandin-specific fluorescence was not found in the primordial follicles, theca externa follicle, granulosa cells of follicles or connective tissue of the ovaries. Luteal cells of the corpora lutea (26 active corpora lutea were examined) showed no fluorescence.

The ovaries of 9 rats at different stages of pregnancy have been studied. The cell types which contained ligandin in non-pregnant rats were also found to be ligandin$^{+}$ in pregnant females. Besides this, ligandin was found in luteal cells of

FIG. 2.

FIG. 3.

FIG. 2 and 3.—Ligandin in the ovaries of rats. Incubation with labelled donkey antibodies after incubation with anti-ligandin antibodies. Fluorescence of the cells of interstitial tissue (IT), atretic follicle cells (AF) and thecal cells persisting around corpora lutea (arrows). Granulosa cells of follicles (GF) and luteal cells (GL) of corpora lutea are dark. 2: × 200; 3: × 60.

Fig. 4.—Ligandin in the ovary of a pregnant rat. Fluorescence of the luteal cells of corpora lutea. × 200.

corpora lutea (Fig. 4) in 7/9 pregnant rats. Twenty-seven active corpora lutea and 17 corpora lutea at the stage of involution were examined in these 9 pregnant rats. All active corpora lutea were ligandin$^+$. Ligandin was absent from all corpora lutea at the stage of involution. In some ligandin$^+$ corpora lutea only single cells contained ligandin. However, equal distribution of ligandin among all luteal cells of the corpus luteum was more often observed. The intensity of the fluorescence of the corpus luteum in pregnant rats was very variable, but always less than the intensity of the fluorescence of interstitial cells. Fluorescence of the nucleus was usually more bright than that of the cytoplasm.

The present immunomorphological findings of ligandin in the gonads expand the previous data (Bannikov *et al.*, 1973; Fleischner *et al.*, 1977) obtained by the immunodiffusion method only. Ligandin has been found in those gonadal cells in which very active steroidogenesis proceeds: Leydig cells, interstitial cells of the ovaries, cells of theca interna follicles and luteal cells in pregnant rats.

It might be suggested that ligandin, identified with glutathione-S-transferase B (Habig *et al.*, 1974; Jakoby *et al.*, 1976) prevents the damage of the steroidogenic active cells caused by high concentrations of steroids. We, however, have no knowledge of any reports on glutathione-S-transferase activity in gonads. It is possible that the function(s) of ligandin, at least in gonadal tissue, is not restricted to detoxication. For example, ligandin might be involved in intracellular transport of haem-containing cytochromes of endoplasmic reticulum (Ketterer *et al.*, 1975).

We thank Professor Ju. M. Vasiliev for encouragement during this work, together with valuable discussions, and Drs M. I. Bronstein and E. A. Ird for consultative help in the morphological examinations.

### REFERENCES

Bannikov, G. A. & Tchipysheva, T. A. (1972) Distribution of azo-dye-binding protein in the organs of rats and mice. *Bull. Exp. Biol. Med. (USSR)*, (in Russian). p. 77.

Bannikov, G. A., Guelstein, V. I. & Tchipysheva, T. A. (1973) Distribution of a basic azo-dye-binding protein in normal rat tissues and carcinogen-induced liver tumours. *Int. J. Cancer*, **11**, 398.

Fleischner, G. M., Robbins, J. & Arias, I. M. (1977) Cellular localization of ligandin in rat, hamster and man. *Biochem. Biophys. Res. Comm.*, **74**, 992.

HABIG, W. H., PABST, M. J., FLEISCHNER, G., GAITMAITAN, Z., ARIAS, I. M. & JAKOBY, W. B. (1974) The identity of glutathione-S-transferase B with ligandin, a major binding protein of liver. *Proc. Nat. Acad. Sci. USA*, **71**, 3879.

JAKOBY, W. B., KETLEY, J. N. & HABIG, W. H. (1976) Rat glutathione-S-transferases: binding and physical properties. In *Glutathione: Metabolism and Function* Eds. I. M. Arias & W. B. Jakoby, Kroc Foundation Series Vol 6, New York: Raven Press. p. 213.

KETTERER, B., ROSS-MANSELL, P. & WHITEHEAD, J. K. (1967) The isolation of carcinogen-binding protein from livers of rats given 4-dimethylamino-azobenzene. *Biochem. J.*, **103**, 316.

KETTERER, B., TIPPING, E., BEALE, D., MEUWISSEN, J. & KAY, C. M. (1975) Proteins which specifically bind carcinogens. *Proc. XI Int. Cancer Congr. Chemical and Viral Oncogenesis*. p. 25.

KIRSCH, R., KAMISAKA, K., FLEISCHNER, G. & ARIAS, I. M. (1975) Structural and functional studies of ligandin, a major renal organic anion binding protein. *J. Clin. Invest.*, **55**, 1009.

LITWACK, G., KETTERER, B. & ARIAS, I. M. (1971) Ligandin: a hepatic protein which binds steroids, bilirubin, carcinogens and a number of exogenous anions. *Nature*, **234**, 466.

SMITH, G. J., OHL, V. S. & LITWACK, G. (1977) Ligandin, the glutathione-S-transferases and chemically induced hepatocarcinogenesis: A review. *Cancer Res.*, **37**, 8.